# Inherent Limitations of Smartphone GNSS Positioning and Effective Methods to Increase the Accuracy Utilizing Dual-Frequency Measurements

**DOI:** 10.3390/s22249879

**Published:** 2022-12-15

**Authors:** Jeonghyeon Yun, Cheolsoon Lim, Byungwoon Park

**Affiliations:** Department of Aerospace Engineering and Convergence Engineering for Intelligent Drone, Sejong University, Seoul 05006, Republic of Korea

**Keywords:** Android, smartphone, GNSS, L1/L5 dual-frequency, SBAS, Xiaomi Mi8

## Abstract

Xiaomi Mi8 with a Broadcom BCM47755 chip, an Android smartphone that supports multi-constellation (GPS, GLONASS, Galileo, BeiDou, and QZSS) and dual-frequency (L1/E1 and L5/E5), was launched in May 2018. Unlike previously released smartphones, it was technically expected to provide robust precise positioning with a fast ambiguity resolution, which led many researchers to be overly optimistic about the applicability of high-accuracy techniques such as real-time kinematic (RTK) systems and precise point positioning (PPP) of smartphones. The global navigation satellite system (GNSS) raw measurement quality of Android smartphones is, however, inherently far lower than that of general GNSS receivers due to their structure, which accordingly makes it difficult for them to be realized. Considering inherent limitations of smartphones such as low-quality antenna, frequent cycle slips, and the duty cycle, a practical strategy including L5 measurements, pseudo-range corrections for L5, and a weighting method is proposed in this paper. The results show that the proposed methods of L5 differential GNSS (DGNSS) and Doppler-based filtering can guarantee a positioning accuracy of 1.75 m horizontally and 4.56 m vertically in an Android device, which is comparable to the performance of commercial low-cost receivers.

## 1. Introduction

In the recent smart mobility era, the use of smartphone location-based services such as remote vehicle driving and mobility location sharing services, as well as navigation and route-finding services, is increasing. For example, Android Auto users can easily use smartphone location-based navigation services in the vehicle by connecting their smartphone to a vehicle that does not have a built-in global navigation satellite system (GNSS) [1]. Because this service relies on the location accuracy of a smartphone installed inside the vehicle rather than a GNSS antenna installed on the vehicle ceiling, incorrect directions often occur. As another example, Tesla recently released a new technology known as Smart Summon, which allows a Tesla vehicle to be called through a smartphone application [2]. It enables a Tesla vehicle to drive itself towards the owner’s location based on the relative position between the vehicle and the smartphone. Since the GNSS of a smartphone cannot provide an accurate position, the owner should be cautious of potential malfunctions such as the target point being set incorrectly. However, the GNSS chipset currently used to calculate the location of smartphones has an error of more than 5–10 m in open-sky and 20–100 m in urban areas with many obstacles, such as buildings. Before Google’s announcement of providing GNSS raw measurements [3], all the location-based products and services were totally dependent on the location accuracy that the chipsets provide. Smartphone and GNSS chipset vendors previously did not allow general users to feed the correction to the module or access the GNSS pseudo-range [4,5], which resulted in smartphones providing horizontal accuracy up to 10 m with a 95% probability [6,7].

After Android GNSS raw measurements were introduced, many researchers worked on assessing the positioning performance of smartphones. However, the low-cost chips in currently commercialized smartphones do not guarantee reliable high-quality positioning results as general GNSS receivers do. Smartphones use low quality linearly polarized (LP) antenna optimized for voice communication. Since it was not right hand circularly polarized (RHCP) specialized for navigation signals, smartphones are very vulnerable to multipath interference. As a result, multipath errors of smartphones are higher than a few tens of meters [3]. To apply high-precision positioning techniques such as RTK and PPP to smartphones, external equipment or various strong constrains are essential. In particular, continuous carrier-phase measurements are difficult to obtain because of the duty-cycle effect. Moreover, conservation time of smartphone PPP is much longer than geodetic receivers due to unstable carrier-phase measurements [3,6].

The launch of the first dual-frequency GNSS chipset, BCM47755 (designed by Broadcom, San Jose, California, USA), was a big opportunity to reduce the performance gap between the smartphones and general GNSS receivers. The BCM47755 was first installed in the Xiaomi Mi8 (designed by Xiaomi, Haidian District, Beijing, China) in May 2018, and supported multi-constellation (GPS, GLONASS, Galileo, BeiDou and QZSS) and dual-frequency (L1/E1 and L5/E5). Recently, researchers tried to apply high-end technologies, utilized in general GNSS receivers, to Android smartphones. Fortunato applied RTK and PPP methods to the Xiaomi Mi8 and achieved horizontal root mean squares (RMS) of 1.17 m and 2.23 m in dynamic positioning, respectively [7]. Since he utilized the code-minus-carrier phase (CMC) observable method, a separate pretreatment process was required to void the vulnerability by interference, multipath, or cycle slip. Despite preprocessing, ambiguity fix was not enabled. Wu investigated the application of PPP in static and kinematic modes using the Xiaomi Mi8 [6]. The convergence time of dual-frequency smartphones in static experiments was 102 min for 1 m and 116 min for 0.2 m, which is far longer than 40 min of general GNSS receivers. A single-frequency PPP strategy-based clock bias estimation method was studied [8], but the matrix size is too large to be used as real-time high-precision smartphone positioning because the state of the filter had to include the integer ambiguities for all satellites.

Common problems in existing studies include interference, multipath, and cycle slip due to the use of carrier-phase. Additionally, carrier ambiguity fix requires very long convergence times and a complex matrix. To overcome these limitations, this study introduces a method to utilize the L5 measurement, which is strong against noise and multipath error. We also suggest a method to provide the L5 Pseudo Range Correction (PRC) for accurate positioning of an Android smartphone without building a new infrastructure or correction messages. In addition, we present a method to use Doppler measurements instead of carrier phase, and it does not require cycle slip monitor and ambiguity determination processes that caused the complexity and frequent discontinuity due to the instability of carrier-phase measurement. It is expected to be practical and economical to use a smartphone itself aligned with existing infrastructures without adding or modifying the device and current systems.

The remainder of this paper is organized as follows. In Section 2, the obstacles to applying precision positioning method to Android smartphones are described. In Section 3, the strategy for reliable accuracy improvement algorithm for Android is introduced. Further, a field test results after implementing the suggested algorithm on the user side is presented and examined in Section 4. Finally, Section 5 presents the discussion and conclusions.

## 2. Obstacles to Applying Precision Positioning Technique to an Android Smartphone

### 2.1. Low Quality of Android Smartphones Antenna

RHCP GNSS antennas are used for commercial geodetic receivers, which are optimized to reject reflected signals. However, LP planar inverted F (PIF) antennas are embedded in smartphones and optimized for voice communication with a lower volume. The vulnerability of Android GNSS raw measurements to multipath interference primarily arises due to the antenna configuration.

The PIF antenna significantly affects the quality of the received GNSS signal. First of all, the multipath and noise of smart devices are much larger than the received signals through general GNSS antennas. Noise and multipath error RMS of Android smartphone L1 code was observed up to 10 m under open-sky and reported over 20 m in a multipath environment. It directly affected the smartphone positioning performance, and an error of 11.65 m in vertical RMS was remained even though all the other errors such as ionospheric and tropospheric delay were removed by applying DGNSS [8,9]. Despite the high elevation angle over 60°, weak signal strength of 27 to 33 dB-Hz C/No were observed with frequent loss-of-locks [9], which are very rare cases for RHCP antennas. In addition, uncertainty of the exact phase center was reported to cause the bias of the phase residuals after double-difference [10].

**Figure 1:**
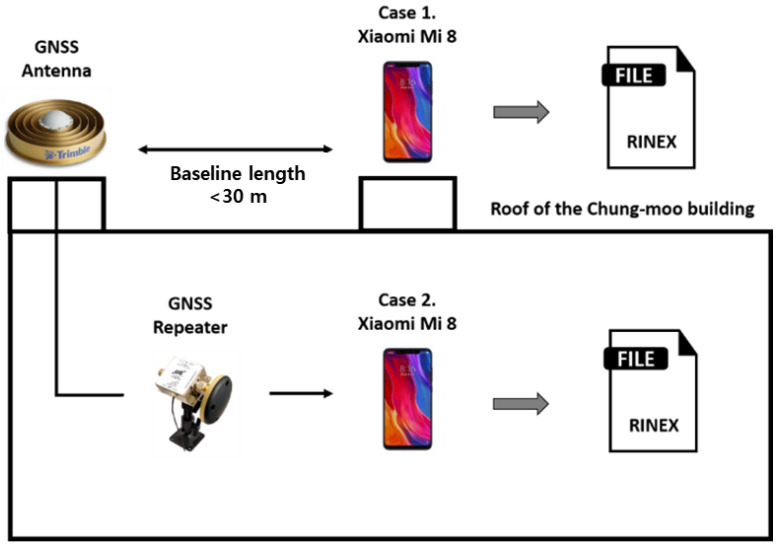
Test configuration for comparison of noise-level with or without GNSS repeater.

To examine the effect of antenna configuration on the smartphone, we conducted a static test, as shown in Figure 1.

Unlike other GNSS receivers, the hardware structure makes it difficult to connect a smartphone directly to a geodetic antenna. GNSS repeaters were used to solve this problem [10,11]. Open-sky GNSS signals were directly re-radiated to one smartphone via a GNSS repeater coupled with a geodetic antenna to eliminate interference from the surrounding environment and amplify the receivable signal strength. We placed a Xiaomi Mi8 Android smartphone to receive signals that were transmitted by a repeater via a Trimble choke ring antenna. The other Mi8 was placed to receive live GNSS signals at the rooftop where the choke ring antenna was implemented. We collected pseudo-range (P) and carrier-phase (Φ) for both cases using the two smartphones at the same period, which are denoted in meters in Equations (1) and (2).

(1) $$P_{r,i}^{s}=d_{r}^{s}-b^{s}+B_{r}+T_{r}^{s}+I_{r,i}^{s}+\varepsilon_{P_{r,i}^{s}},$$

(2) $$\Phi_{r,i}^{s}=d_{r}^{s}-b^{s}+B_{r}+T_{r}^{s}-I_{r,i}^{s}+\lambda_{i}^{s}N_{r,i}^{s}+\varepsilon_{\Phi_{r,i}^{s}},$$

where d, b, B, T, I, λ, N and ε denote the geometric range from satellite to ground receiver, satellite clock bias, receiver clock bias, tropospheric delay, ionospheric delay, carrier wavelength, integer ambiguity, and measurement noise, respectively. The subscript, r, denotes each test scenario, Open-sky for case 1 and Repeater for case 2. The superscript, s and subscript, i, denote the s-th satellite and type of carrier-frequency, respectively.

The combination of code and carrier described in Equation (3) enables all the geometric and ionospheric terms removed, so that there are only integer ambiguity and noise terms remained in $R_{r}^{s}$.

(3) $$R_{r}^{s}=\left(P_{r,L_{1}}^{s}-P_{r,L_{5}}^{s}\right)+\left(\Phi_{r,L_{1}}^{s}-\Phi_{r,L_{5}}^{s}\right),$$

**Figure 2:**
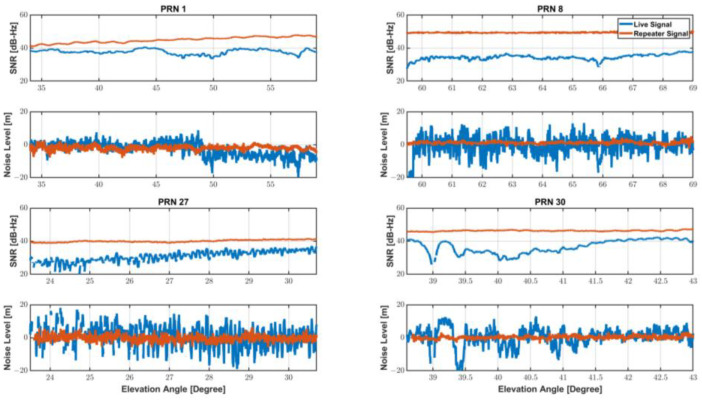
SNR and Noise-level of Live and Re-radiated signal.

In this case, since the integer ambiguity has a constant value that does not change unless a cycle-slip occurs, the integer ambiguity can be estimated by taking the average value. Therefore, it is easy to compare the pseudo-range noise levels of live and re-radiated signals after removing the integer ambiguity from Equation (3), since L1 and L5 pseudo-range noise terms are dominant over carrier-phase noise. Table 1. and Figure 2. shows the measurement noise level with the SNR measured for the two cases. The signal to noise ratios (SNR) of the smartphone live signals were generally lower than re-radiated signal via a choke-ring antenna, and accordingly noise levels of the live signals were far higher.

The smartphone observed the signals passing through the GNSS repeater with an SNR value of 40 dB-Hz or higher. In contrast, the SNR of the PRN 8 was observed around 30 dB-Hz when a smartphone receiving live signal and did not exceed 40 dB-Hz despite a high elevation angle of 60° or more. Signal SNR observed by a commercial GNSS receiver generally increases as the elevation angle increases. The clear relationship between elevation and SNR, however, is not found in smartphones equipped with low-cost GNSS antenna. To make matters worse, as can be seen from PRNs 27 and 30, SNR and noise fluctuations due to multipath were significantly severe to smartphone measurements.

In summary, the Android GNSS measurement had much a larger noise level and a lower correlation to the satellite elevation than that of a typical GNSS receiver, and is very vulnerable to multipath error [12]. Therefore, we should find ways to reduce the multipath effect on the measurement results and devise a new noise modeling [13] and weighting method appropriate for noise characteristics of smartphone GNSS modules.

### 2.2. Unstable and Discontinuous Measurements

**Figure 3:**
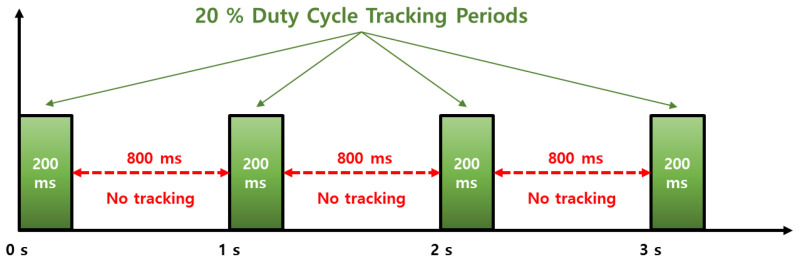
Duty-cycle versus time.

**Figure 4:**
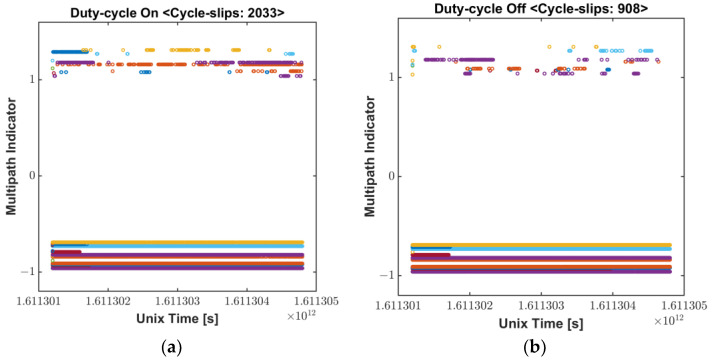
Cycle-slip Flag: (**a**) Duty-cycle On; (**b**) Dufy-cycle Off.

Smartphone vendors prioritize to maximize power consumption, and accordingly, a duty cycle technique is implemented to maintain a low power consumption because continuously operating the GNSS chipset drains the battery [7,14]. The navigation chip on the smartphone tracks the signal for 200 ms/s and is dormant for 800 ms/s when the duty cycle is on, which limits the continuous acquisition of measurements, as shown in Figure 3. While this feature would not degrade code measurement quality, it does have a significant impact on carrier-phase measurements. Without continuous tracking, several cycle slips may occur between two consecutive measurements, which severely limit the use of advanced phase-based precise positioning techniques such as RTK or PPP. To cope with the hardware limitation for the precise positioning, a function to turn off the duty-cycle, called “Force full GNSS measurements,” was added at the Android 9 Pie update in 2018. However, executing the duty-cycle off mode does not completely remove the cycle slips, as shown in Figure 4.

Even though the function of the duty-cycle de-activation was reported to reduce the cycle slips found in the duty-cycle on mode by more than 50%, it is not so perfect as to prevent all the measurements from being slipped. As described in Section 2.1 above, PIF antennas optimized for voice signals are highly affected by multipath errors. Although “Force full GNSS measurements” may be effective to reduce cycle slips caused by duty-cycle function, it is difficult to completely remove all the cycle slips, therefore a substantial number of cycle-slips still remain. Because of this instability and discontinuity of Android carrier-phase, the ambiguity determination process in precise positioning should be often initialized, which results in long convergence time in PPP. Wu et al., reported that the convergence time within 0.2 m of a smartphone was up to 272 min, which was longer than twice of a geodetic receiver converging time, 116 min [6].

**Figure 5:**
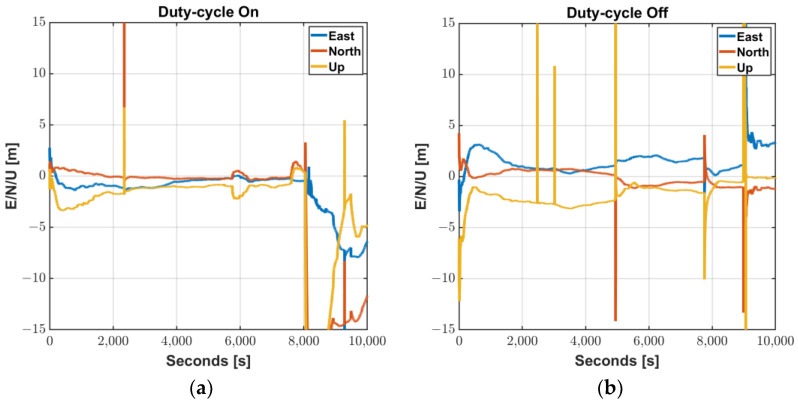
RTKLIB Processing Results of Mi8 L1/L5 GPS Measurements: (**a**) Duty-cycle On; (**b**) Duty-cycle Off.

In RTK cases, frequent unstable measurements on a smartphone and their misdetection hinder the ambiguity to be fixed or often cause wrong ambiguity determination, resulting in float solutions for a long period or large position errors. We processed GPS L1/L5 measurement results obtained from a Mi8 to examine how much the duty-cycle off function actually contributes to making the carrier-based positioning robust. GPS observables for the two modes were logged from two Mi8 devices, and their results, processed by a RTKLIB software package [15], were compared in Figure 5. The commonly used RTK algorithm, which could provide cm-level accurate position to commercial GPS receivers, is not valid to Android measurements for both duty-cycle modes. Integer ambiguities of the carrier-phase measurements were rarely determined through all the test periods. Subsequently, position errors in each direction were barely found within 1 m. Frequent initialization and wrong position fixes, which are typical results caused by undetected cycle slips, were found in both modes. In conclusion, the “Force full GNSS measurements” function can effectively eliminate the cycle-slip caused by the duty-cycle, however, a lot of cycle-slip remains due to the structural limitations of the Android smartphone. Even with the duty-cycle turned off, errors of more than 10 m at five points and non-continuous carrier-phase measurements still do not achieve centimeter-level accuracy.

### 2.3. Carrier-Phase Is Not Available on All Android Smartphones

Smartphones with Android Nougat operating systems (OS) and above allow access to Google’s application program interfaces (APIs) that provide GNSS raw measurements of pseudo-range, carrier-phase, and Doppler measurements. Although Android OS is up-to-date and the dual-frequency carrier-phase available chip is included in devices, it is totally up to the manufacturer whether or not each measurement is provided to the public.

Even though code measurements filtering based on carrier-phase is essential to improving the positioning accuracy, recently released smartphones mostly do not support carrier-phase measurements. Galaxy S21 Z-Fold and Z-Flip series, flagship models of Samsung that ranked first with 19.0% market share (Table 2, [16]), do not provide carrier phase measurement to users. Xiaomi, the third ranker of the market and the manufacturer of the dual-frequency carrier-phase enabled smartphone Mi8, stopped providing carriers in its latest release, Mi 9 (see Table 3) [17].

There are few smartphones remaining in the market that carrier-phase based precise positioning methods can be applied to. Instead, dual-frequency, multi-constellation, and Doppler capabilities has become more general. Therefore, it is necessary to find a way to improve the accuracy of smartphones more universally considering the market trends.

## 3. Strategy for Reliable Accuracy Improvement of Android Smartphone Positioning

### 3.1. Usage of the L5 Code Measurements

L5 band signals such as GPS L5 and GAL E5 have a high chipping rate, 10 times higher than that of L1 frequency signals [18]. In addition, it includes a pilot channel, which can lead to a total integration time. These new features of L5/E5 signals are less prone to multipath errors; therefore, they provide inherent noise and multipath mitigation capabilities [19].

**Figure 6:**
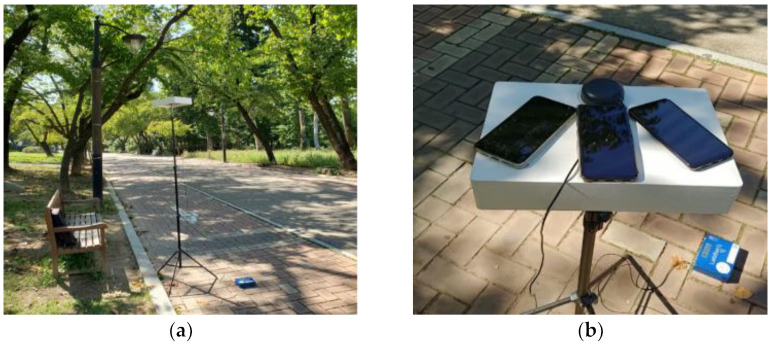
Experiment place: Children’s Grand Park near the Sejong University: (**a**) experimental environment; (**b**) experimental equipment: Android smartphones.

To compare the multipath error characteristics of L1 and L5 pseudo-range measurements, we constructed a signal reception experiment for 30 min (2019/09/18 04:40:00~05:09:59 UTC) in Children’s Grand Park, Seoul, Korea. There were many obstacles such as trees, as shown in Figure 6.

In Equation (4), an ionospheric-free residual is obtained by combining code and phase measurement, which contains multipath error and noise components.

(4) $$MP_{r,i}^{s}+\varepsilon_{P_{r,i}^{s}-\Phi_{r,i}^{s}}=P_{r,i}^{s}-\Phi_{r,i}^{s}-\frac{2\cdot f_{j}^{2}}{f_{i}^{2}-f_{j}^{2}}\left(\Phi_{r,i}^{s}-\Phi_{r,j}^{s}\right)\;\;\;\;+\lambda_{i}^{s}N_{r,i}^{s},$$

**Figure 7:**
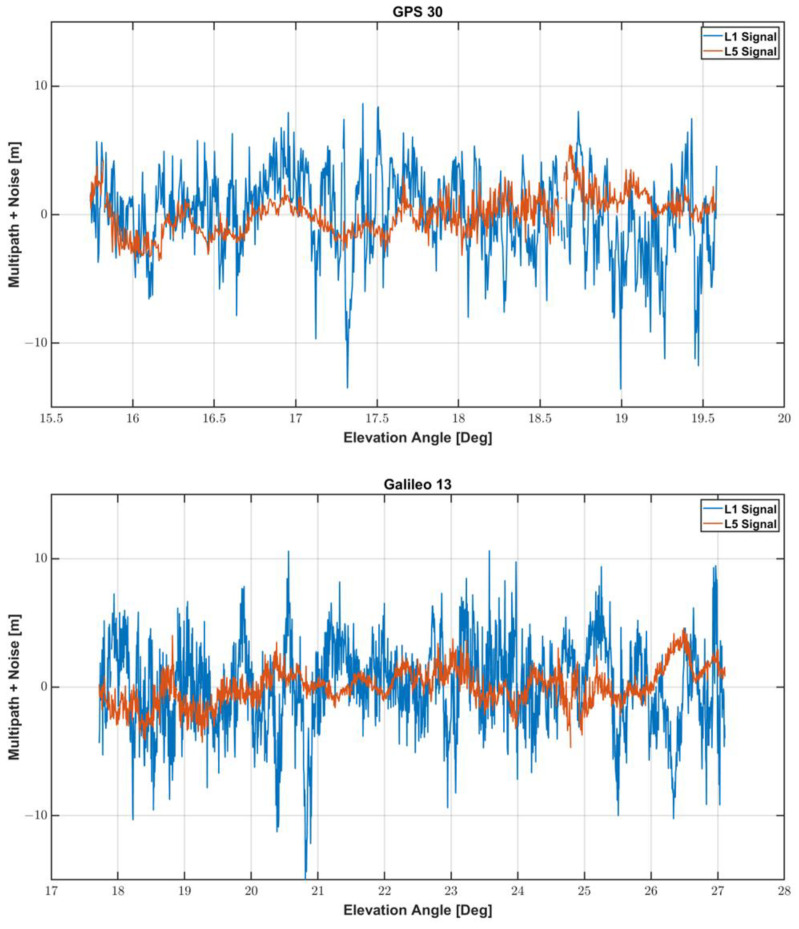
Multipath error and noise at GPS 30 and GAL 13 satellites in L1 and L5 Frequency.

We assessed multipath errors of L1 and L5 frequencies by comparing the results when GPS 30 and GAL 13 satellites were employed. The two satellites were observed from the elevation angles smaller than 30° in the southeast, whose signals are heavily affected by nearby obstacles. Figure 7 shows the multipath error and noise for the two satellites. As summarized in Table 4, the RMS and 95% percentile errors of L1 frequency measurements for the two multipath-affected satellites were about 3.4 and 6.5 m, respectively, which is about 2.4 times larger than those of L5 frequency. The maximum multipath of L5 was about 5 m, and it was reduced by 60 to 70% compared to the maximum error of L1.

Considering the low noise level and multipath characteristics of the L5 signal, the use of L5 code measurements can achieve higher positioning results than the conventional L1 single-frequency positioning.

### 3.2. Enhancing Available L5 Signals by Adding Weighted L1 Signals

**Figure 8:**
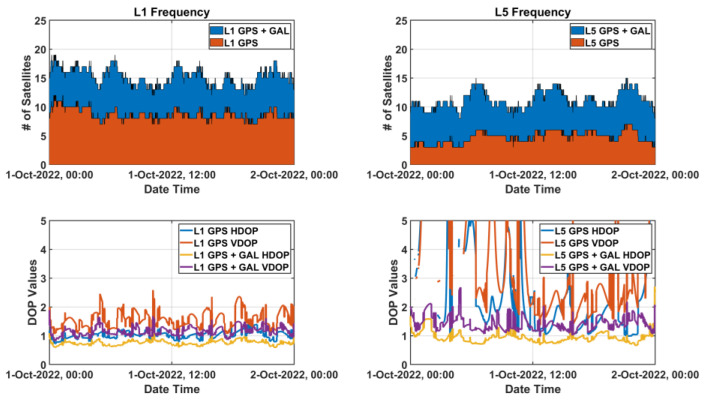
Available satellites status observed at SOUL reference station, Korea on 1 October 2022.

Noise and multipath performance of L5 measurement is better than that of L1 measurement. In the case of GPS, however, only 17 out of total 31 satellites currently transmit the L5 signal [20]. The number of available L5 GPS signals is frequently observed to be less than four, as shown for about 24 h (1 October 2022 00:00:00~24:00:00 UTC) in Figure 8, which means that smart devices cannot understand their positions for approximately half a day. Although GPS-Galileo multi-constellation might enable the L5 positioning, the increased DOP values due to a bad satellite geometry cannot guarantee high accuracy performance of positioning. At about 23:50, when the L5 satellite’s Horizontal DOP (HDOP) was the highest, the L5 DOP was three times larger than that of the L1 DOP, and at about 04:50, when the L5 satellite’s Vertical DOP (VDOP) was the highest, the L5 DOP was twice larger than that of the L1 DOP. Moreover, the satellite geometry might be worse than expected because it is reported that fewer Galileo satellites were usually observed by Android smartphones than general commercial receivers, and their signal tracking quality was relatively poor [6]. Therefore, L5-only positioning performance is not guaranteed to provide better results than L1-only because of the satellite geometry, although L5 signal itself has a better performance than L1.

Nevertheless, the excellent L5 signal performance must be utilized, and thus a method to overcome the low number of available L5 signals and poor geometry must be considered. In this study, we suggest adding L1 signals to L5-only positioning and giving different weight by noise-level to each frequency to take advantage of L5 signals.

To implement L1/L5 GPS/Galileo algorithm, observation matrix (H) for each constellation and frequency is defined in Equation (5).

(5) $$\left[\begin{array}{c} H_{L_{1}}^{GPS}\\ H_{L_{1}}^{GAL}\\ H_{L_{5}}^{GPS}\\ H_{L_{5}}^{GAL} \end{array}\right]=\left[\begin{array}{ccccc} E_{L_{1}}^{GPS} & \vec{1} & \vec{0} & \vec{0} & \vec{0}\\ E_{L_{1}}^{GAL} & \vec{0} & \vec{1} & \vec{0} & \vec{0}\\ E_{L_{5}}^{GPS} & \vec{0} & \vec{0} & \vec{1} & \vec{0}\\ E_{L_{5}}^{GAL} & \vec{0} & \vec{0} & \vec{0} & \vec{1} \end{array}\right],$$

$\vec{e}\left({\vec{e}}_{x},\;{\vec{e}}_{y},\;{\vec{e}}_{z}\right)$ is a line-of-sight unit vector from a user to each satellite, E is a group of $\vec{e}$ vectors for each constellation and frequency, which is defined as m-by-3 matrix, $E_{L_{1}}^{GPS}$ represents the number of GPS L1 signals, m. Since user clock bias of each constellation and frequency is different, H-matrix is defined as $\left(m+n+o+p\right)$-by-7 matrix, and n, o, and p stands for the number of Galileo (GAL) L1 signals, GPS L5 signals, and GAL L5 signals, respectively, where $\vec{1}$ and $\vec{0}$ are vectors of ones and zeros, each.

As previously shown, GNSS signal tracking of smart devices is often unstable even at high elevations, and its SNR fluctuation is generally large. Therefore, it is recommended to use a noise modeling function of both elevation angle and signal strength when setting a weighting matrix. One common method for weighting the pseudo-range for the s-th satellite based on its elevation angle (el) and the SNR is Equation (6) [21]. The coefficient $k_{j}$ for the L1 and L5 frequency has been added to the equation, which need to be assigned based on the noise characteristics for the frequency.

(6) $${\left(\sigma_{j}^{s}\right)}^{2}=k_{j}^{2}\cdot \frac{10^{\left(-0.1\times SNR^{s}\right)}}{\sin^{2}\left(el^{s}\right)},$$

According to Section 3.1, noise at L1 frequency was observed to be 3 m-level, and noise at L5 frequency was observed to be 1 m-level. Thus, we set $k_{1}$ and $k_{5}$ as 3 and 1, respectively. In addition, we assumed that the noise levels of GPS and Galileo were similar. The weighting matrix for both L1 and L5 signals in Equation (7) enables L5 measurements with good signal performance to be used as a primary source of the positioning and several L1 measurements to provide good satellite geometry.

(7) $$R=\left[\begin{array}{cccc} R_{L_{1}|GPS} & 0 & 0 & 0\\ 0 & R_{L_{1}|GAL} & 0 & 0\\ 0 & 0 & R_{L_{5}|GPS} & 0\\ 0 & 0 & 0 & R_{L_{5}|GAL} \end{array}\right]$$

where

$$R_{j|GPS}=\left[\begin{array}{cccc} {\left(\frac{1}{\sigma_{j|GPS}^{1}\;}\right)}^{2} & 0 & \cdots & 0\\ 0 & {\left(\frac{1}{\sigma_{j|GPS}^{2}\;}\right)}^{2} & \cdots & 0\\ \vdots & \vdots & \ddots & \vdots \\ 0 & 0 & \cdots & {\left(\frac{1}{\sigma_{j|GPS}^{m}\;}\right)}^{2} \end{array}\right],\;R_{j|GAL}=\left[\begin{array}{cccc} {\left(\frac{1}{\sigma_{j|GAL}^{1}\;}\right)}^{2} & 0 & \cdots & 0\\ 0 & {\left(\frac{1}{\sigma_{j|GAL}^{2}\;}\right)}^{2} & \cdots & 0\\ \vdots & \vdots & \ddots & \vdots \\ 0 & 0 & \cdots & {\left(\frac{1}{\sigma_{j|GAL}^{m}\;}\right)}^{2} \end{array}\right]$$

### 3.3. L5 Pseudo-Range Correction Generation

DGNSS and Wide Area DGNSS (WADGNSS) are typical methods for mitigating code measurement errors [19,20]. Pseudorange received from two GNSS receivers located in close proximity (e.g., within a few hundred kilometers) will contain the same atmospheric errors. To eliminate this, users can mitigate common errors by using base stations with precise and known locations. Since the base receiver knows the actual geometric position between the GNSS satellites and the receiver, it can use this to create corrections from differences in pseudorange measurements received from the satellites. Correction information can be applied in real time on site using radio signals or post-processed using specialized processing software. A similar system that transmits corrections from satellites instead of ground-based transmitters is called a Wide Area Augmentation System (WAAS) or WADGNSS. A Satellite-Based Augmentation System (SBAS), sometimes used synonymously, can include satellite systems implemented in many parts of the world, such as EGNOS, MSAS, QZSS, and GAGAN.

In a similar way for general GNSS receivers, GNSS common errors, such as satellite-related and atmospheric errors, must be mitigated to improve the smartphone position accuracy. The correction messages for the code measurements usually extract or model the error for each satellite itself as defined in Radio Technical Commission for Maritime services (RTCM) Special Committee (SC)-104 version 2 [22] or Radio Technical Commission for Aeronautics (RTCA) DO-229 [23]. On the other hand, Multiple Signal Messages (MSM) that support multi-frequency carrier phase are close to observables [24,25] as defined in RTCM SC-104 version 3. While the bandwidth for the code measurement corrections is bound to several hundred bps [26], the carrier phase corrections cost high-rate transmission data has a bandwidth of at least 9600 bps [27]. However, the overall performance of accuracy and initialization of the smartphones carrier phase positioning has been reported to not be so outstanding as typical commercial receivers. Carrier-phased based positioning employed to smartphones should wait stationary over several or even up to tens of minutes in order to get cm-level results [28]. Furthermore, the long initialization time could not guarantee accurate positioning of cm-level accuracy, which is an added disadvantage [29] and often results in wrong ambiguity resolution or convergence. Therefore, code-based DGNSS positioning is more practical in Android smartphone than phase-based one, and L1/L5 dual frequency for multi-constellation GNSS would enhance the smartphone DGNSS performance.

The errors included in the L5 code measurement should be also mitigated by using correction message since the L5 signal is expected to contribute significantly to the accuracy improvement of the code-based positioning, as discussed in the previous section. Unfortunately, neither DGNSS nor Satellite Based Augmentation System (SBAS) currently provide correction for the frequency other than L1, and this strategy is not able to be applied to smartphones with the existing infrastructures [30]. Moreover, it is unreasonable and impractical to build new infrastructures for L5 signals to improve smartphone positioning accuracy. Therefore, we need to propose a new method to mitigate L5 code measurement errors of smartphones based on the existing infrastructures.

Multi-constellation GNSS errors due to the various sources, i.e., the tropospheric (T) and ionospheric (I) error and satellite-related error (δR) would be effectively mitigated if only the nearby reference station supports as many GNSS constellations as the smart device. PRC and Carrier-Phase Correction (CPC) for DGNSS are generated by Equations (8) and (9), respectively.

(8) $$PRC_{L_{1}}=d_{r}^{s}-P_{r,L_{1}}^{s}-b^{s}+B_{r}=-T_{r}^{s}-I_{r,L_{1}}^{s}-\delta R_{r}^{s}-\varepsilon_{P_{r}^{s}},$$

(9) $$CPC_{L_{1}}=d_{r}^{s}-\Phi_{r,L_{1}}^{s}-b^{s}+B_{r}=-T_{r}^{s}+I_{r,L_{1}}^{s}-\delta R_{r}^{s}-\lambda_{L_{1}}^{s}N_{r,L_{1}}^{s}-\varepsilon_{\Phi_{r}^{s}},$$

where superscript s, subscript $L_{1}$, and subscript r represent the satellite id, L1 frequency, and a reference station, respectively. $d_{r}^{s}$ is the geometric range between the s-th satellite and the reference station, $P_{r,L_{1}}^{s}$ and $\Phi_{r,i}^{s}$ are L1 frequency pseudo-range and carrier-phase. The satellite clock bias and receiver clock bias are denoted as $b^{s}$ and $B_{r}$, respectively.

Range Rate Correction (RRC) accompanied with PRC are usually calculated by differentiating CPC rather than PRC, as shown in Equation (7) [31], since the time difference of PRC is far noisier than the CPC rate or Doppler [32]. By the time differencing of Equation (10), the integer ambiguity term $\left(\lambda_{L_{1}}^{s}N_{r,L_{1}}^{s}\right)$ has been removed so that only GNSS common error related terms remain in RRC.

(10) $$RRC_{L_{1}}={\dot{CPC}}_{L_{1}}=-{\dot{T}}_{r}^{s}+{\dot{I}}_{r,L_{1}}^{s}-\delta {\dot{R}}_{r}^{s}-{\dot{\varepsilon }}_{\Phi_{r}^{s}},$$

The generated PRC and RRC can mitigate the errors in pseudo-range and Doppler $\left(D_{u,L_{1}}^{s}\right)$ measurements of a rover station, respectively, as Equations (11) and (12) show.

(11) $$P_{u,L_{1}{}_{correct}}^{s}=P_{u,L_{1}}^{s}+PRC_{L_{1}}\approx d_{u}^{s}-b^{s}+B_{u}+\varepsilon_{P_{ru}^{s}},$$

(12) $$D_{u,L_{1}{}_{correct}}^{s}=D_{u,L_{1}}^{s}+RRC_{L_{1}}\approx {\dot{d}}_{u}^{s}-{\dot{b}}^{s}+{\dot{B}}_{u}+\varepsilon_{D_{ru}^{s}},$$

Current DGNSS standard [25] defines PRC and RRC for only L1, and no DGNSS reference station is providing DGNSS services for L2 or L5. Although L5 signals are far more beneficial for smart device positioning than L1, implementing a new L5 DGNSS infrastructure for only a smart device service is not economical. Moreover, it is impossible without revision of standards for L5.

Therefore, we suggest a practical solution for generating L5 PRC correction in a user side using current L1 PRC and SBAS message based on current DGNSS infrastructures as well as standards. The only difference in PRC between L1 and L5 is ionospheric error due to the dispersive signal characteristics [33], and thus we can get $Ic_{L_{5}}$ for the L5 signals based on the L1 ionospheric correction from the SBAS messages.

(13) $$IC_{L_{5}}=\gamma \cdot IC_{L_{1}}$$

where $\gamma ={\left(\frac{L_{1}\;Frequency}{L_{5}\;Frequency}\right)}^{2}$.

Since the SBAS messages, unlike PRC of DGNSS, enable generating ionospheric correction based on its pierce point, it can compensate for the L5 PRC difference for all the visible satellites as presented in Equation (13). Most GNSS receivers can compensate for the ionospheric error difference by themselves using Equations (14) and (15) because they include built-in SBAS functions.

(14) $$PRC_{L_{5}}=PRC_{L_{1}}-\left(\gamma -1\right)IC_{L_{1}}=-T_{r}^{s}-I_{r,L_{1}}^{s}-\delta R_{r}^{s}-\left(\gamma -1\right)IC_{L_{1}}-\varepsilon_{P_{r}^{s}}\approx -T_{r}^{s}-I_{r,L_{5}}^{s}-\delta R_{r}^{s}-\varepsilon_{P_{r}^{s}},$$

(15) $$RRC_{L_{5}}=RRC_{L_{1}}+\left(\gamma -1\right){\dot{IC}}_{L_{1}}=-{\dot{T}}_{r}^{s}+{\dot{I}}_{r,L_{1}}^{s}-\delta {\dot{R}}_{r}^{s}+\left(\gamma -1\right){\dot{IC}}_{L_{1}}-{\dot{\varepsilon }}_{\Phi_{r}^{s}}\approx -{\dot{T}}_{r}^{s}+{\dot{I}}_{r,L_{5}}^{s}-\delta {\dot{R}}_{r}^{s}-{\dot{\varepsilon }}_{\Phi_{r}^{s}},$$

Finally, the compensated PRC and RRC are fed to the pseudo-range and Doppler measurement as described in Equations (16) and (17). Allocating separate L5 frequency reference stations or related infrastructures and messages is not required by the correction generation.

(16) $$P_{u,L_{5}{}_{correct}}^{s}=P_{u,L_{5}}^{s}+PRC_{L_{5}}\approx d_{u}^{s}-b^{s}+B_{u}+\varepsilon_{P_{ru}^{s}},$$

(17) $$D_{u,L_{5}{}_{correct}}^{s}=D_{u,L_{5}}^{s}+RRC_{L_{5}}\approx {\dot{d}}_{u}^{s}-{\dot{b}}^{s}+{\dot{B}}_{u}+{\dot{\varepsilon }}_{D_{ru}^{s}},$$

### 3.4. Code Filtering Using Doppler Measurements

Our last proposal to improve the smart device positioning accuracy is using Doppler measurements instead of carrier-phase to filter the code measurements. Commonly used filtering methods such as Hatch-filter and Kalman-filter usually filter the code measurements using carrier-phase measurements because carrier-phase noises are much smaller than that of code measurements [34,35].

However, many Android smartphones do not support carrier-phases as shown in Table 3. In addition, the ambiguity of carrier-phases on Androids is not kept constant even when the duty-cycle is turned off, which causes frequent cycle slips without any notice or float ambiguity, thus remaining unfixed. The Doppler equivalent of the time derivative of carrier-phase is available from all Android devices on the market and free from the cycle slip.

**Figure 9:**
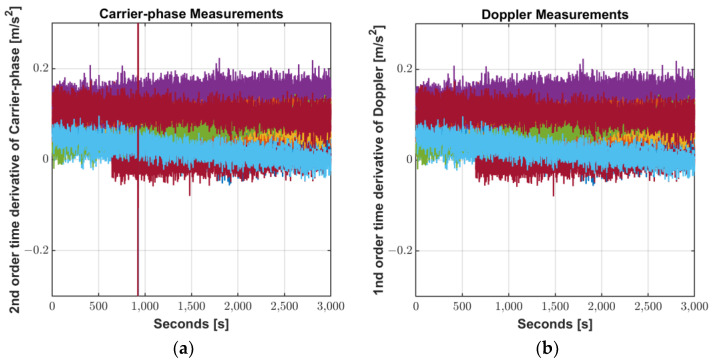
Noise level of measurements: (**a**) 2nd order time derivative of Carrier-phase; (**b**) 1st order time derivative of Doppler.

In commercial geodetic receivers, Doppler measurements have a higher noise level than that of the carrier-phase time differenced measurements [9], however, the noise levels of both measurements are so similar on the Mi8. It is unclear as the chipset vendor does not provide detailed algorithms for measurement generating process, but the smartphone’s chipset seems to provide carrier phase measurements generated in a different manner than commercial receivers. Figure 9 compares the noise level of the 2nd order time derivative of carrier-phase measurements against 1st order time derivative of Doppler measurements obtained from Xiaomi Mi8. Excluding the abnormal jumps due to carrier-phase discontinuity, the discrepancy is small with a magnitude of approximately 6.4 × 10^−8^ m.

Sharp jumps due to unexpected cycle-slips of the phase shown in the green dashed line box in Figure 9 can cause filter initialization and divergence, but this can be prevented for Doppler without cycle slip. In addition, as mentioned in Section 2.3, not all smartphones support carrier-phase measurements, but all Doppler measurements are supported. Therefore, a practical filtering method to smart devices L1/L5 code measurements should be performed with Doppler rather than phase difference.

## 4. Smartphone Positioning Algorithm Implementation and Field Test Results

### 4.1. Configuration of Field Test

**Figure 10:**
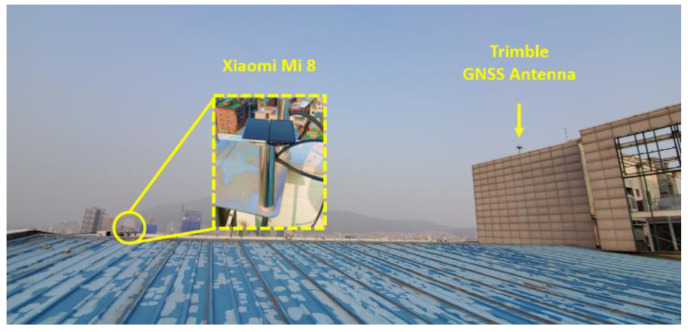
Test Environment at the Roof of the Chung-moo building in Sejong University.

A static field test was carried out for 50 min (1 April 2019 08:00:00~08:50:00 UTC) on the rooftop of the Chung-moo building, Sejong University, Seoul, Korea to obtain raw measurements of dual-frequency smartphone Xiaomi Mi8. Figure 10 shows the actual signal-receiving environment of the static test site. Two Mi8 were installed 30 m apart from the reference station of Trimble NetR9 for DGNSS.

During the test, eight GPS satellites (PRN 1, 7, 8, 11, 16, 18, 27, 30) and three Galileo satellites (PRN 15, 27, 30) were observed under the open-sky. Among them, four GPS (PRN 1, 8, 27, 30) and three Galileo (PRN 15, 27, 30) satellites provided L5 frequency signals. To use SBAS message for the ionospheric compensation, MTSAT Satellite Augmentation System (MSAS) PRN 129 message in #RAWSBASFRAMEA format was transmitted from a Novatel flexpak 6 receiver and decoded. Corrections for L1/L5 DGNSS, $PRC_{L_{1}}$, and $RRC_{L_{1}}$ were obtained by the transmitted RTCM v2 message from the Trimble NetR9 receiver. $PRC_{L_{5}}$ and $RRC_{L_{5}}$ were generated after compensating the ionospheric error by the transmitted #RAWSBASFRAMEA from the flexpak 6 receiver as described in Equations (14) and (15).

### 4.2. L1/L5 DGNSS Results

**Figure 11:**
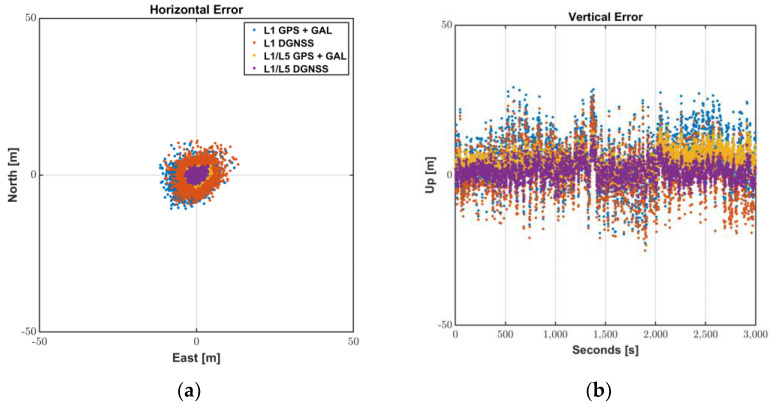
L1/L5 DGNSS positioning results: (**a**) Horizontal Error; (**b**) Vertical Error.

Figure 11 and Table 5 show the L1 and L1/L5 DGNSS positioning results of Mi8 smartphone. The masks of elevation angle and SNR were set to 20° and 20 dB-Hz, respectively. L1 DGNSS used the L1 PRC and RRC generated for all available GNSS by the existing in infrastructure. Then, we estimated ionospheric error for each signal of other GNSS using ionospheric vertical delays at the grid points surrounding its pierce point. Subsequently, in L1/L5 DGNSS, the L5 PRC and RRC compensated by the received SBAS message according to Equations (14) and (15) were fed to the Android L5 GNSS measurements.

The vertical average error, which was larger than 5 m for L1 GPS + GAL, was reduced to 1.3 m by the compensated L1/L5 DGNSS PRC generation. Moreover, better signal quality by adding L5 satellites made it possible for the Android user get the horizontal position of 2.7 m in 95%. Providing good satellite geometry by L1 multi-constellation GNSS and enabling L5 signals to be available by compensating L5 DGNSS correction would be more beneficial to enhance the position availability and accuracy of users, especially with low satellite visibility in urban or mountainous areas.

Therefore, the Android user can successfully mitigate L5 GNSS error as well as L1 error using existing L1 reference stations and received SBAS messages, without adding infrastructures or modifying the related correction message standards.

### 4.3. L1/L5 Doppler-Based Kalman-Filter Results

Kalman filter is a recursive filter that estimates the state of a linear dynamics system based on random noise statistical characteristics. A two-step approach to prediction and update can reduce GNSS positioning errors from an optimization point of view. In this study, we defined a state of Kalman filter at time k, 12 × 1 vector of $X_{k}$, which consists of position, velocity, clock bias, clock drift, and Inter-signal Bias, as shown in Equation (18).

(18) $$X_{k}={\left[\begin{array}{cccccccc} \vec{x} & B_{L_{1}}^{GPS} & B_{L_{1}}^{GAL} & \vec{\dot{x}} & {\dot{B}}_{L_{1}}^{GPS} & {\dot{B}}_{L_{1}}^{GAL} & ISB^{GPS} & ISB^{GAL} \end{array}\right]}^{T}$$

where $\vec{x}$ is three-dimensional position vector, and $\vec{\dot{x}}$ is its velocity vector. The user clock biases and user clock drifts for GPS and Galileo satellites of L1 frequency are denoted as $B_{L_{1}}^{GPS}$, $B_{L_{1}}^{GAL}$, ${\dot{B}}_{L_{1}}^{GPS}$ and ${\dot{B}}_{L_{1}}^{GAL}$, respectively. Inter-signal Bias, ISB, represents the difference of L1/L5 frequency, which is assumed constant during the session. Dynamics of the state were modeled as the first order state prediction based on the relationship between $\left[\vec{x}\;B\right]$ and $\left[\vec{\dot{x}}\;\dot{B}\right]$, while the ISB was set as a random walk [36]. The noise level ratio of L5 signals to L1, described in Section 3.1, was considered to define covariance matrix of the observables, $R_{k}$, for the measurement update. The measurement vector and $\left(m+n+o+p\right)\times 1$ vector of $Z_{k}$ are obtained from observables for L1 and L5. Unlike Kalman filters for general GNSS receivers, carrier-phase observables were replaced with Doppler to reliably filter the Android pseudo-range noise as described in Equation (19).

(19) $$Z_{k}^{s}=\left[\begin{array}{c} \left(P_{u,L_{1_{correct}}}^{s}-e\cdot R^{s}+b^{s}\right)\\ \left(P_{u,L_{5_{correct}}}^{s}-e\cdot R^{s}+b^{s}\right)\\ \left(D_{u,L_{1_{correct}}}^{s}-e\cdot {\dot{R}}^{s}+{\dot{b}}^{s}\right)\\ \left(D_{u,L_{5_{correct}}}^{s}-e\cdot {\dot{R}}^{s}+{\dot{b}}^{s}\right) \end{array}\right],$$

**Figure 12:**
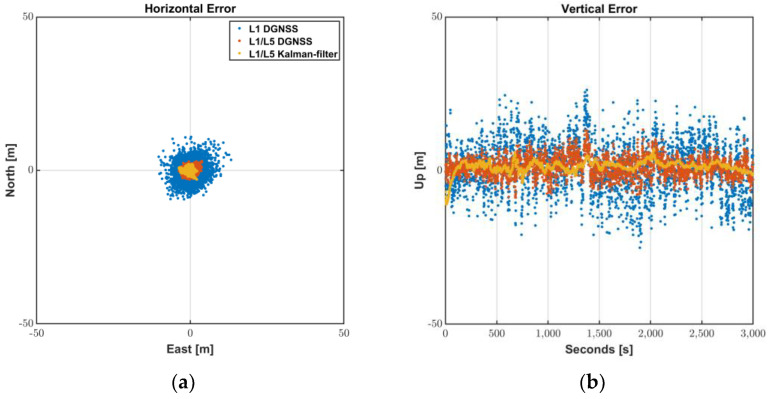
L1/L5 Kalman-filter positioning results: (**a**) Horizontal Error; (**b**) Vertical Error.

The results of L1/L5 Kalman filter are shown in Figure 12 and summarized in Table 6. The positioning accuracy was improved by 20 to 40% after applying the suggested Doppler-based filter so that the RMS values were reduced to 1.2 m horizontally and 2.3 m vertically. During 95% of the periods of the test sessions, the Android devices were able to provide positions with horizontal accuracy of 2.3 m, which shows that L1/L5 DGNSS was properly filtered by the suggested filter construction.

## 5. Conclusions

Since Google’s announcement of providing GNSS raw measurements in 2016, many expected that accurate location information with only several centimeters of error would be provided soon from the palm of the hand. To meet the expectations of the public, many researchers have made efforts to apply PPP or RTK technology to Android devices, and recently published papers have presented the results of feasibility studies. However, considering the smart device manufacturing technologies, market status, and implemented infrastructure for improving positioning accuracy, it might be difficult to realize most of the introduced technologies.

This study discussed why overly optimistic use of high-accurate applications such as RTK or PPP with Android raw measurements alone are difficult to be implemented. LP antenna optimized for voice communication reduces the quality of the GNSS signal and makes the signals be more vulnerable by multipath. Duty-cycle technique for battery saving obstructs measuring continuous carrier-phase, which makes ambiguity estimation of RTK and PPP meaningless. Even when the duty-cycle is turned off, frequent initializations of carrier tracking have been reported. The absence of infrastructures to improve L5 pseudo-range measurements would be a major obstacle when a real service is considered.

To overcome the limitation of smart devices and solve the problems in the real world, practical methods for improving the positioning were proposed as follows:

1. Using weighted L5 code measurements with less-weighted L1 measurements is effective in reducing positioning errors due to the noise and multipath.
2. Feeding L5 PRC after compensating L1 PRC currently in service is efficient way to mitigate the GNSS measurement errors without implementing new infrastructures for L5 service.
3. In the case of the filtering method using Doppler measurements, it can be used even in smartphones that do not support carrier-phase measurements. It also has the advantage of not having to detect an ambiguity cycle sip.

The L5 weighting and Doppler-based filtering method can guarantee that the Android devices provide the positions with an accuracy of 2.32 m horizontally and 4.19 m vertically for most of the time (95%). In addition, the Doppler-based filtering was helpful to increase the reliability of the location information so that the maximum error was bounded within 5 m.

The methods proposed in this paper are of great practical significance as they can be applied to any Android device on the market using the existing infrastructure or service; moreover, they could guarantee assured achievable performance. Therefore, we expect our proposed methods to be a useful and practical solution for not only founding the basis of future Android location-related 4th industrial technologies, but also for improving the location performance of smart devices themselves.

## Figures and Tables

**Table 1:** SNR and Noise-level with or without GNSS repeater results.

PRN	Signal Type	SNR [dB-Hz]	Noise Level [m]	
		MEAN	RMS	95%
GPS 1	Live	37.8412	2.5963	5.0824
	Repeater	44.9657	1.3853	2.6775
GPS 8	Live	33.1333	4.7425	9.2270
	Repeater	48.9788	0.8384	1.6925
GPS 27	Live	32.9258	6.8558	13.3884
	Repeater	41.7745	2.0198	3.9691
GPS 30	Live	37.0858	6.2621	14.0582
	Repeater	45.8212	1.0534	2.0153

**Table 2:** Worldwide smartphone sales to end users by vendor in 2021 (Gartner, March 2022 [16]).

Vendor	2021 Units (Thousands of Units)	2021 Market Share (%)
Samsung	272,327.5	19.0%
Apple	239,239.1	16.7%
Xiaomi	189,305.4	13.2%
OPPO	138,242.1	9.6%
Vivo	136,011.3	9.5%
Others	458,733.9	32.0%
Total	1,433,859.4	100.0%

**Table 3:** Android devices that support raw GNSS measurements (Android Developers [17]).

Model	Android Version	ADR (Carrier-Phase)	L5 Frequency	Global System
Samsung Galaxy S20/S21 (Snapdragon)	12.0	No	Yes	GPS, GLO, GAL, BDS, QZS
Xiaomi Mi9	9.0	No	Yes	GPS, GLO, GAL, BDS, QZS
Samsung Galaxy Note 10	9.0	No	Yes	GPS, GLO, GAL
LG G8 ThinQ	9.0	No	Yes	GPS, GLO, GAL
One Plus 7	9.0	No	Yes	GPS, GLO, GAL
Pixel 3	9.0	No	Yes	GPS, GLO, GAL, BDS

**Table 4:** Multipath error and noise results.

Results		MAX [m]	STD [m]	RMS [m]	95% [m]
GPS 30	L1 Frequency	13.5913	3.3551	3.3532	6.4155
	L5 Frequency	5.4317	1.5012	1.5004	2.9514
GAL 13	L1 Frequency	15.0005	3.4839	3.4829	6.6878
	L5 Frequency	4.7303	1.4258	1.4254	2.9438

**Table 5:** Statistics of L1/L5 DGNSS positioning results.

Positioning Results		MEAN [m]	STD [m]	RMS [m]	95% [m]
L1 GPS + GAL	Horizontal	1.7001	4.4629	4.7750	9.3116
	Vertical	5.0637	8.3905	9.7988	19.0114
L1 DGNSS	Horizontal	0.2916	4.3820	4.3909	8.6137
	Vertical	1.0586	8.0881	8.1558	15.8754
L1/L5 GPS + GAL	Horizontal	0.7607	1.4872	1.6703	3.3183
	Vertical	4.6150	3.9226	6.0564	11.1689
L1/L5 DGNSS	Horizontal	0.2991	1.4023	1.4336	2.7209
	Vertical	1.2965	3.1716	3.4259	6.8974

**Table 6:** Statistics of L1/L5 Kalman-filter positioning results.

Positioning Results		MEAN [m]	STD [m]	RMS [m]	95% [m]
L1 DGNSS	Horizontal	0.2916	4.3820	4.3909	8.6137
	Vertical	1.0586	8.0881	8.1558	15.8754
L1/L5 DGNSS	Horizontal	0.2991	1.4023	1.4336	2.7209
	Vertical	1.2965	3.1716	3.4259	6.8974
L1/L5 KF	Horizontal	0.2449	1.1304	1.1564	2.3188
	Vertical	1.2906	1.9074	2.3028	4.1931
